# Chromosome-specific sequencing reveals an extensive dispensable genome component in wheat

**DOI:** 10.1038/srep36398

**Published:** 2016-11-08

**Authors:** Miao Liu, Jiri Stiller, Kateřina Holušová, Jan Vrána, Dengcai Liu, Jaroslav Doležel, Chunji Liu

**Affiliations:** 1 CSIRO Agriculture and Food, 306 Carmody Road, St Lucia, QLD 4067, Australia; 2 Triticeae Research Institute, Sichuan Agricultural University, Wenjiang, Chengdu 611130, China; 3 Institute of Experimental Botany, Centre of the Region Haná for Biotechnological and Agricultural Research, Šlechtitelů 31, CZ-78371 Olomouc, Czech Republic; 4 School of Plant Biology, The University of Western Australia, Perth, WA 6009, Australia

## Abstract

The hexaploid wheat genotype Chinese Spring (CS) has been used worldwide as the reference base for wheat genetics and genomics, and significant resources have been used by the international community to generate a reference wheat genome based on this genotype. By sequencing flow-sorted 3B chromosome from a hexaploid wheat genotype CRNIL1A and comparing the obtained sequences with those available for CS, we detected that a large number of sequences in the former were missing in the latter. If the distribution of such sequences in the hexaploid wheat genome is random, CRNILA sequences missing in CS could be as much as 159.3 Mb even if only fragments of 50 bp or longer were considered. Analysing RNA sequences available in the public domains also revealed that dispensable genes are common in hexaploid wheat. Together with those extensive intra- and interchromosomal rearrangements in CS, the existence of such dispensable genes is another factor highlighting potential issues with the use of reference genomes in various studies. Strong deviation in distributions of these dispensable sequences among genotypes with different geographical origins provided the first evidence indicating that they could be associated with adaptation in wheat.

Bread wheat (*Triticum aestivum* L., 2n = 6x = 42, AABBDD genome) is the staple food for 30% of the world’s population. The bread wheat genome is huge (~17 gigabases) and complex (consisting of 3 homoeologous subgenomes) and more than 80% of the highly redundant genome consists of repeated sequences^1^,^2^. To overcome the complexity of the bread wheat genome, the International Wheat Genome Sequencing Consortium (IWGSC) adopted a strategy of physical mapping and sequencing individual chromosomes and chromosome arms of the genotype Chinese Spring (CS). The IWGSC has completed a survey of the gene content and composition of all 21 wheat chromosomes and identified 124,201 gene loci, with more than 75,000 positioned along the chromosomes^3^. Sequencing work on chromosome 3B is the most advanced and a nearly complete pseudomolecule sequence (~774.4 Mb) for this chromosome is now available, and sequence annotation predicted 7,264 genes along this chromosome^4^. The pseudomolecule sequence would significantly facilitate research on any traits controlled by genes on this chromosome.

Many genes of agronomic importance are located on wheat chromosome 3B. A total of 121 QTLs for 50 different traits on this chromosome were reported when describing the 3B pseudomolecule^4^. Of the numerous QTLs conferring Fusarium crown rot (FCR) resistance^5^, the one with the largest effects is located on this chromosome^6^,^7^. Working toward the cloning of gene(s) underlying FCR resistance, near isogenic lines (NILs) have been obtained for this locus^8^. Transcriptome analysis was conducted against several pairs of these NILs and genes with differential expressions in the targeted QTL region were identified^9^. A NIL-derived population has also been used in mapping the locus to an interval of 0.7 cM covering a physical distance of about 1.5 Mb^10^. As part of our effort in cloning genes underlying the FCR locus on this chromosome, we isolated and sequenced chromosome 3B from a FCR resistant line CRNIL1A. By comparing the sequences obtained from this chromosome with those of CS, a large number of CRNIL1A fragments likely missing or significantly altered in CS were detected. To evaluate the possible relationship between these CS-missing fragments and adaptation, distributions of these CS-missing fragments among genotypes from different parts of the world were assessed. Implications of main results obtained in these analyses are discussed in this paper.

## Results

### CRNIL1A sequence reads obtained and those map to the 3B pseudomolecule of CS

A total of 36.6 Gb (about 122.4 million) raw sequences were obtained from the sorted chromosome 3B of CRNIL1A. As the length of this chromosome was about 886 Mb^2^, the CRNIL1A sequences represent a sequencing depth of 41 times for this chromosome. Over 74.0 million high-quality reads were obtained after trimming. About 87.9% (~65.0 million) of these reads were successfully mapped to the 3B pseudomolecule of CS with a mean read coverage of 17.1. The mapped reads covered a total of 692.7 Mb (or 89.5%) of the CS 3B pseudomolecule (Table 1).

### CRNIL1A reads not found on the CS 3B pseudomolecule

Matching sequences for about 9.0 million CRNIL1A sequence reads were not found on the 3B pseudomolecule of CS (Table 1). These reads were *de novo* assembled into 221,705 contigs. Of them, 304 (0.14%) appeared to be non-plant sequences. The remaining 221,401 clean contigs represented a total length of 101.2 Mb and 139,035 of them were longer than 300 bp.

**Figure 1:**
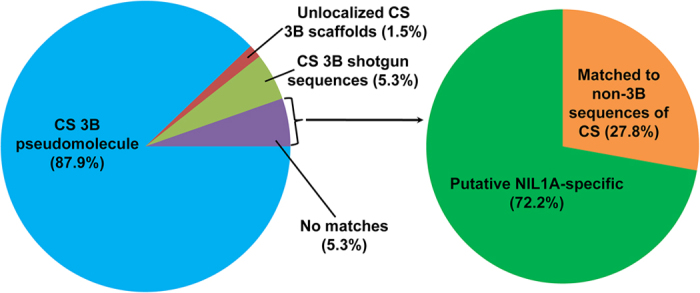
Classification of sequence reads obtained from flow-sorted chromosome 3B of CRNIL1A in comparison with available sequences of the hexaploid wheat genotype Chinese Spring (CS). Matches of the ~74.0 million high-quality reads to various components of CS 3B sequences are shown on the left and those that could not be mapped to the CS 3B on the right.

Detailed analyses were carried on 3,563 of these clean contigs with a length of 2 kb or longer. These contigs covered a total length of 13.7 Mb. Five hundreds and two of these contigs (covering 1.6 Mb in total) found matches from non-3B sequences of CS and they likely represent contaminations in the sorted 3B chromosome (Fig. 1). Matches for 1,127 of these contigs were not found from the available sequences of any CS chromosomes including those of the single flow-sorted chromosome 3B^11^. These 1,127 putative CRNIL1A-specific contigs covered a total length of 4.3 Mb (Fig. 1). Considering that these putative CRNIL1A-specific contigs may contain short sequences with high similarity with those of CS, we randomly selected and analysed 100 of them against the CS shotgun sequences using a 70% sequence identity. Matching sequences of 50 bp or longer were removed and the remainders were considered putative sequences missing in CS. The putative CS-missing sequences accounted for about 26.1% of the total sequences represented by these putative CRNIL1A-specific contigs. Assuming such sequences are evenly distributed across the hexaploid wheat genome, CRNIL1A sequences missing on chromosome 3B of CS would be about 8.3 Mb, and as much as 159.3 Mb of the CRNIL1A sequences could be missing in the whole genome of CS.

### Validation of the putative CRNIL1A-specific sequences

**Figure 2:**
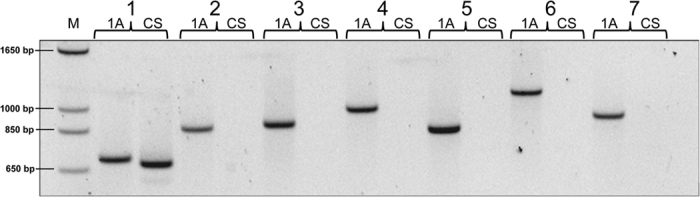
Validation of CRNIL1A-specific contigs against CRNIL1A (1A) and Chinese Spring (CS) by PCR amplification. M = marker; 1 = contig_10984; 2 = contig_55; 3 = contig_14304; 4 = contig_18594; 5 = contig_2968; 6 = contig_4048–1; and 7 = contig_7552.

These sequences can be classified into two groups. One contains contigs for which no matches were found in the CS sequences. A total of 43 pairs of primers were designed based on 39 of such contigs. A single pair of primers was initially designed for each of these contigs. Five of them failed to generate specific fragments from CRNIL1A (smear or no product at all). The failed amplifications could be due to several reasons including the nature of the targeted sequences, and low quality of sequences or assemblies. PCR products from the other 33 primer pairs were all in agreement with the results of the sequence comparison between these two genotypes. They produced fragments with expected sizes from CRNIL1A but generated no product from CS (Fig. 2). In addition to the expected fragments, the primer pairs targeting *contig_10984* also generated a product from the total DNA of CS (Fig. 2), indicating that the available CS sequences are incomplete. To further confirm that the missing sequences in CS were genuine, a second pair of primers was then designed for four of these contigs. As anticipated, none of them generated any products from CS (Supplementary Table S1).

The 34 primer pairs which generated discrete products from CRNIL1A were assessed against the parents of the segregating populations. Among them, 11 generated fragments differing in size between parents for one or both of the populations. Linkage analyses showed that all of the polymorphic fragments generated by these primer pairs mapped to chromosome 3B (Supplementary Figs S1 and S2).

**Figure 3:**
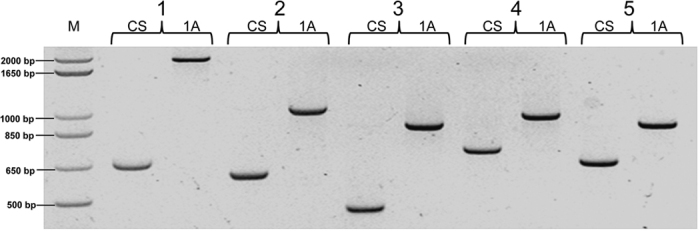
Validation of long insertions in CRNIL1A (1A) compared with sequences of Chinese Spring (CS) by PCR amplification. M = marker; 1 = contig_46411; 2 = contig_57900; 3 = contig_128179; 4 = contig_97492; and 5 = contig_163046.

The other group of CRNIL1A-specific sequences contains those large-sized insertions for which matching sequences were not found in CS. The difference between these insertions and those of CRNIL1A-specific contigs is that sequences flanking these insertions were found between both of these genotypes. To detect such insertions, all sequence reads from sorted chromosome 3B of CRNIL1A were *de novo* assembled. About 80.5% of the reads were assembled into 469,671 contigs with an average length of 704 bp. The longest 1,000 contigs were selected and compared with the CS 3B pseudomolecule. This comparison detected 50 putative insertions of 50 bp or longer in size. Primers targeting 24 of these insertions were designed (Supplementary Table S3). Nineteen of them generated only fragments with correct sizes from both CS and CRNIL1A (Fig. 3). In addition to fragments with anticipated sizes from these two genotypes, two of these primer pairs also amplified an extra fragment each from CRNIL1A (Supplementary Table S3). The remainder three primer pairs failed to generate any specific fragments from either CS or CRNIL1A. Again, the failed amplifications could be due to the nature of the targeted sequences or qualities of the sequences or assemblies in concern.

### Distribution of CRNIL1A-specific sequences among hexaploid wheat genotypes with different geographical origins

**Figure 4:**
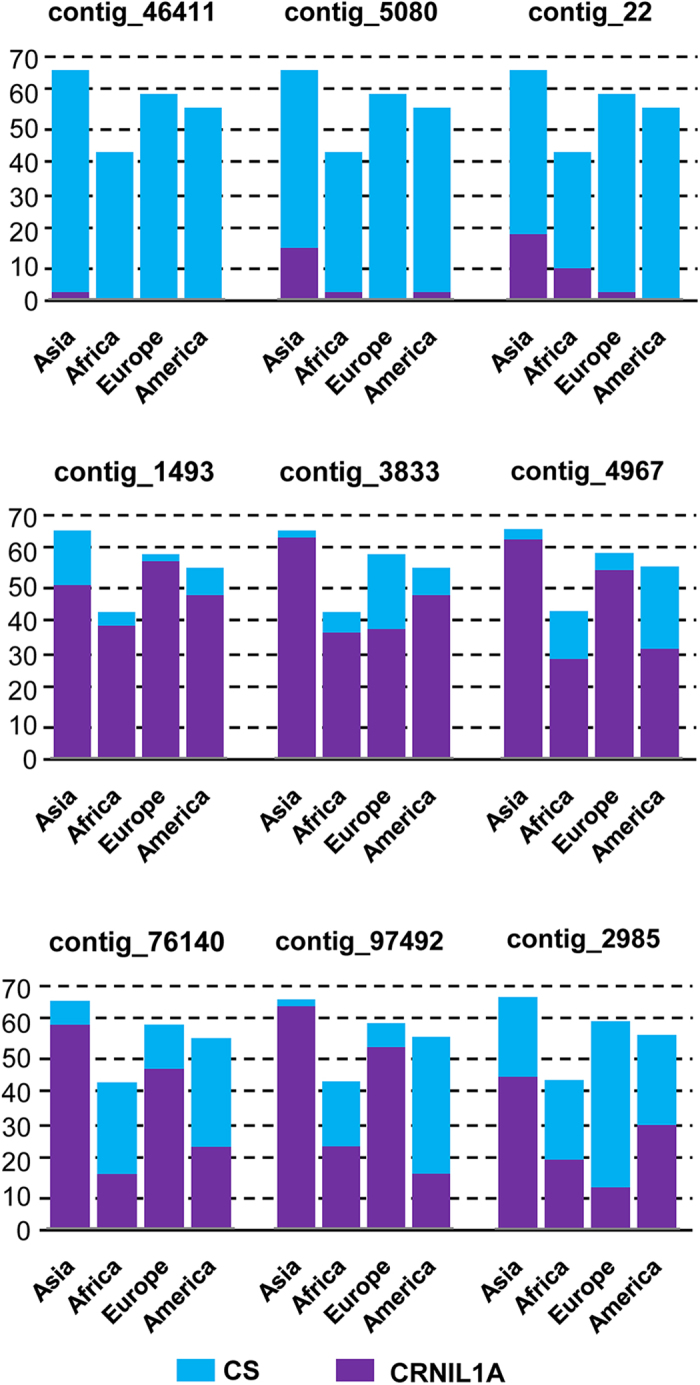
Distributions of the Chinese Spring-type (CS) and CRNIL1A-type (NIL1A) alleles among 226 bread wheat accessions originated from the four different regions worldwide. Y-axis shows the numbers of genotypes from each of the four geographical regions.

Primers targeting 33 of the CRNIL1A-specific contigs and 19 of those large-fragment insertions were then used to assess the distributions of these CRNIL1A-specific sequences among genotypes from different parts of the world. Primers targeting 56 of such sequences were assessed against 226 hexaploid bread wheat genotypes with different geographical origins. Based on the sizes of their PCR products, most of these genotypes can be classified as either CRNIL1A-type or CS-type (Supplementary Table S4) for each of the primer pairs assessed. It may not be surprising that none of these 56 primer pairs generated similar numbers of CRNIL1A- and CS-types for genotypes from each of the regions assessed. However, 58% of these primers consistently detected higher numbers of one type over the other for all of the regions: 19 of them detected more CS-types while another 14 detected more ‘CRNIL1A-type from genotypes belonging to each of the four regions (Fig. 4; Supplementary Table S4). It was also clear that genotypes from Asia showed higher ratio of ‘CS-type’ (Supplementary Fig. S3). Surprisingly, primers targeting *contig_46411* and *contig_163046* detected the CS-type only in genotypes of Asia (Supplementary Table S4).

### Functions of the putative CRNIL1A-specific contigs

**Figure 5:**
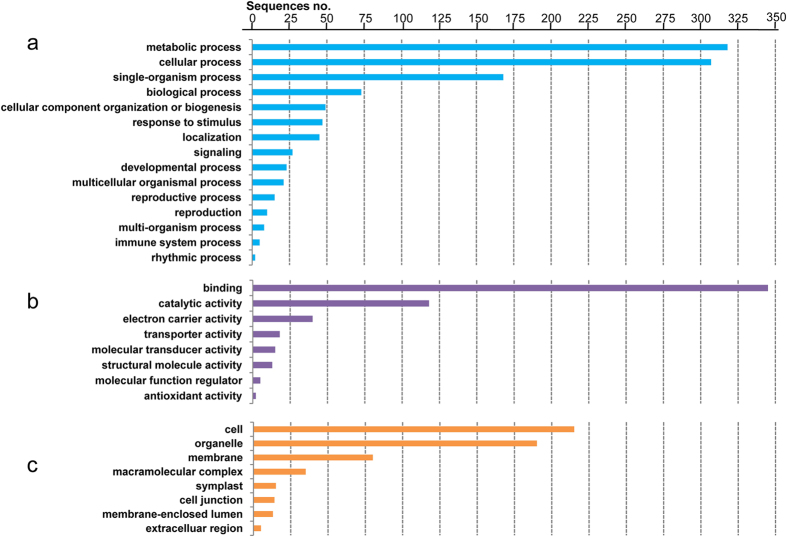
Gene Ontology classification of genes represented by putative CRNIL1A-specific contigs. Assignments of Gene Ontology terms for all sequences based on significant hits among various plant species are summarized into three main GO categories: (**a**) biological process, (**b**) molecular function, and (**c**) cellular component.

A functional annotation analysis was conducted against the 1,127 putative CRNIL1A-specific contigs with 2 kb or longer in length. Significant BLASTX hits to amino acid sequences on GenBank non-redundant (NR) database were not detected for 417 (37.0%) of these contigs. For the other 710 contigs, 211 (18.7%) could not be linked to any Gene Ontology entries or could not obtain an annotation assignment; and 499 (44.3%) were successfully annotated and mapped to one or more of the three organizing principles of GO classifications. Gene functions represented by these contigs are not evenly distributed in any of the GO classes: the most abundant groups represented were metabolic process and cellular process in the biological process class, binding was the most represented GO term in the molecular function class, and cell and organelle were strongly biased for in the class of within cellular component (Fig. 5).

### Hexaploid wheat transcripts not present in CS

To further examine the extent of dispensable genes in hexaploid wheat, published RNA reads from 26 accessions of hexaploid wheat genotypes were assessed. The initial Trinity assembly produced a total of 574,795 transcripts belonging to 344,953 potential genes. After filtering out those putative artefacts of transcript assemblies, 231,126 (≥500 bp) transcripts with a total length of 254.2 Mb were identified for further analysis.

To improve the reliability of novel sequence identification, 8,826 potential transcripts derived from commensal organisms, lab contaminants and all non-plant origin were removed. The remaining 222,300 transcripts were aligned to the CS genome sequences and gene models to identify alleles or paralogs. Of them, 209,926 (94.4%) were successfully mapped to the CS genome. Following clustering to remove duplicates, 9,573 unique transcripts were detected from the 12,374 (5.6%) transcripts not mapped to the CS genome (Supplementary Fig. S4).

To verify the facticity of these transcripts not in CS, we compared them with sequences of wheat and its close relatives. This comparison showed that 290 or 3% of them aligned to wheat EST sequences, 131 or 1.4% aligned with CRNIL1A-specific contigs, 2,119 or 22.1% aligned well with the sequences of the hexploid wheat genotype W7984, and 958 or 10% of them aligned well with novel genomic contigs from 16 bread wheat varieties. When compared with sequences from the close relatives of wheat, 2,222 or 23% of them were found in the durum wheat variety Cappelli, 2,177 or 23% were detected from the durum variety Strongfield, 1,461 or 15.3% were detected from *Aegilops speltoides*, 1,381 or 14.4% were found in *A. sharonensis*, 1,168 or 12.2% of them were found in *Triticum monococcum*, 1,464 or 15.3% were found in *T. urartu*, 1,353 or 14.1% were found in *A. tauschii*. In total, 4,396 or 45.9% of these non-CS transcripts were detected in wheat and its close relatives (Supplementary Fig. S5). BLASTx alignment of the 9,573 novel transcripts against the GenBank NR protein database showed that 6,584 or 68.8% of them had significant hits with a cut-off of 1E-6. A proportion of the remainder non-CS transcripts could be artefacts from sequencing or sequence assembly. They could also contain untranslated regions, noncoding RNA or sequences not containing a protein domain. GO term associations most frequently observed within these novel transcripts were metabolic process, cell associated, and nucleotide binding domains (Supplementary Fig. S6).

## Discussion

The genotype CS has been used worldwide as the reference base in wheat genetics and genomics. Enormous resources have been used to generate a reference genome based on this genotype by the international community^1^,^2^,^3^,^4^,^12^. We demonstrated in the study reported here that a large number of hexaploid wheat fragments are likely missing or significantly altered in the CS genome. Results from the assessment of RNA sequences from 26 hexaploid wheat genotypes further support the notion that dispensable genes are common in hexaploid wheat. Together with those extensive intra-13 and interchromosomal rearrangements in CS^14^,^15^, the existence of these dispensable genes is another factor highlighting potential issues with the use of a single reference genome for any species. In regarding to work related to genome sequencing, we need to be cautious in ordering and orientating sequence contigs based on comparative approaches as different individuals may have different translocations and inversions. We also need to be aware that the widely used method of whole genome resequencing based on a single reference genome^16^,^17^,^18^,^19^ would not only miss large-sized variants but also other types of dispensable elements which could be important and account for large portions of various genomes including microbes^20^,^21^,^22^,^23^,^24^ and plants^25^,^26^,^27^,^28^,^29^,^30^,^31^,^32^.

Previous work in various bacterial and fungal genomes showed that some of the dispensable genes are associated with adaptation^20^,^24^,^33^,^34^. Recent studies from maize^28^,^30^,^32^, soybean^29^ and rice^31^ indicate that this also seem the case in plants. Results from this study showed that this is also likely the case in wheat as a large proportion of the non-CS sequences assessed were not randomly distributed among genotypes with different geographical origins. The distributions of some of these sequences were so extreme that they could only be detected from genotypes belonging to a certain geographical region. It would be of interest to find out the exact functions of these sequences and their effects in breeding varieties for a given environment.

It is important to note that the CS-missing fragments reported in this study represent only a portion of the dispensable genome in hexaploid wheat. Only fragments larger than 50 bp were targeted and they were detected by comparing a single pair of genotypes only. It is not unreasonable to predict that additional sequences missing in CS would be detected if different genotypes were used for comparison. Further, it is also very likely that, compared with other genotypes, CS must also contain some unique fragments. Considering that we still do not have a high-quality reference genome for wheat yet despite of the efforts having been used on the single genotype CS^3^,^4^,^12^, generating only a few more reference genomes for species such as hexaploid wheat would be expensive and yet not provide a good picture of the wheat genome variability. Nevertheless, we need to be aware of the potential issues associated with the use of one or a few reference genomes.

Integrating those insertions identified from CRNIL1A into the CS 3B pseudomolecule can be easily achieved based on sequences flanking each of them. However, determining the relative locations of the CRNIL1A-specific fragments can be more challenging. Linkage analyses based on markers targeting these sequences showed that all polymorphic fragments generated by the 11 pairs of primers assessed were located on the linkage group of chromosome 3B. This is not surprising considering that the sequences obtained were all derived from isolated 3B chromosome and that the proportion of non-3B chromosome sequences detected from such a library is usually small^11^. Further, detailed analysis of the CRNIL1A sequences should also facilitate the effort in integrating those unlocalized CS 3B scaffolds^4^ into the 3B pseudomolecule.

Functional analysis showed that genes represented by the CRNIL1A-specific sequences are not randomly distributed among the various GO function groups (Fig. 5). It is not clear whether the non-random distribution among genes represented by these sequences among the functional groups is caused by the fact that the genotype CRNIL1A was a derivative of *T. spelta* as all of the sequences selected for validation were detected from two or more additional bread wheat genotypes assessed. Considering that CS is very different from modern varieties and that previous studies suggests strong links between dispensable genes and important traits^26^,^27^,^28^,^29^,^30^,^31^,^32^, establishing linkages between these missing fragments and the unique phenotype of CS could be particularly rewarding in detecting genetic variations for characteristics of agronomic importance.

## Methods

### Plant materials

The hexaploid wheat line CRNIL1A was used for sequencing in this study. This line was developed from a backcross Janz*2/CSCR6. Janz is a commercial bread wheat variety and CSCR6 is a *T. spelta* line with high level of CR resistance^8^. CRNIL1A genome fragments missing in hexaploid wheat cv. Chinese Spring (CS) were further analysed against a panel of 226 hexaploid wheat genotypes representing wheat from Asia, Africa, Europe and America (Supplementary Table S1). In addition, two segregating populations were also employed to determine chromosome locations of selected fragments missing in CS. One of the populations consisted of 92 recombinant inbred lines derived from the cross Lang/CSCR6^7^, and the other is a doubled haploid population consisting of 153 lines derived from the cross Batavia/Ernie^6^.

### Flow sorting, library construction and Illumina sequencing of chromosome 3B from the hexaploid wheat genotype CRNIL1A

Aqueous suspensions of mitotic metaphase chromosomes were prepared from synchronized root tip cells of the CRNIL1A line according to Vrána *et al.*^35^ and stained with DAPI (2 μg/ml). The chromosome 3B was flow-sorted using FACSAria SORP flow cytometer (BD Biosciences, Santa Clara, USA) equipped with UV laser (355 nm, 100 mW).

Chromosome 3B was sequenced in two runs on Illumina MiSeq instrument (Illumina Inc., San Diego, CA, USA). For the first sequencing experiment, 25,000 chromosomes representing 50 ng of DNA were sorted twice into 0.5-ml PCR tube containing 40 μl sterile deionized water. Chromosomal DNA was amplified according to Šimková *at al.*^36^, yielding 3.6 μg DNA in each sample. The DNA was purified by Ampure XP beads, the two samples were combined and a sequencing library was prepared from 2 ug DNA using TruSeq DNA PCR-Free sample preparation kit (Illumina) according to manufacturer’s instruction. However, DNA was sheared by Bioruptor Plus (Diagenode, Liege, Belgium) and size selection was modified to achieve insert size about 1,000 bp. For the second sequencing experiment, nine samples of 30,000 chromosomes each were sorted into 0.5-ml PCR tubes containing 40 μl sterile deionized water. DNA was purified, pooled and sequencing library was generated using Nextera DNA Sample preparation kit (Illumina) according to the manufacturer’s instruction. Insert size of the second library was about 500 bp.

Concentration of both sequencing libraries was assessed by KAPA Library Quantification Kit for Illumina (Kapa Biosystems, Woburn, USA). MiSeq Reagent Kit v3 (Illumina) was used for sequencing each library in a single run to produce pair end reads with length of 2 × 300 bp. The first library was clustered to a density of 1339 K/mm2, the second one to 1066 K/mm2.

Raw reads were trimmed using a SolexaQA package 2.2^37^ with minimum Phred 10 quality value of 30 and minimum length of 100 bp. All reads obtained in this work were deposited in the National Center for Biotechnology Information (NCBI) with the accession number of PRJNA310378.

### Mapping CRNIL1A sequence reads to the CS 3B pseudomolecule

The 3B pseudomolecule (traes3bPseudomoleculeV1) was downloaded from https://urgi.versailles.inra.fr/download/wheat/3B4. Trimmed sequences of CRNIL1A were aligned to the CS chromosome 3B pseudomolecule by CLC Genomics Workbench 8.0 with the following settings: mismatch cost (2), insertion cost (3) deletion cost (3), length fraction (0.9) and similarity fraction (0.9).

### CRNIL1A sequence reads that could not be mapped to CS 3B pseudomolecule

All reads which could not be mapped to the CS 3B pseudomolecule were *de novo* assembled using the CLC Genomics Workbench with the following settings: minimum contig length (200), mismatch cost (2), insertion cost (3), deletion cost (3), length fraction (0.95), and similarity fraction (0.5).

Contigs assembled from these reads were first checked by DeconSeq^38^ to remove sequence contamination under stringent parameters of identity ≥90% and coverage ≥5%. Retained contigs were analysed against the unlocalized 3B scaffolds of CS (https://urgi.versailles.inra.fr/download/wheat/3B/)^4^ using the BLAST+blastn algorithm with an E-value threshold 10^−10^. To identify CRNIL1A-specific contigs, those with ≥90% identity and over 75% of the contig length were all removed. The remaining contigs were further compared with all available sorted chromosome shotgun sequence (CSS) of CS (http://www.wheatgenome.org/)^3^, the whole genome shotgun sequences of CS (TGACv1) (http://plants.ensembl.org/), and the contigs obtained from a multiple displacement amplification of the DNA from single flow-sorted 3B chromosome of CS^11^. Different from those for chromosome 3B, the minimum sequence identity requirement for comparing with other chromosomes of hexaploid wheat was decreased to 80%. Considering that some contigs of CS shotgun sequences were short, additional 9 hits were visually inspected to determine if the sequence length of the best hit was less than 75% of the length for a given CRNIL1A contig. Those contigs which did not meet these criteria were classified as CRNIL1A -specific. In estimating the total length of CRNIL1A sequences missing in CS, 100 of CRNIL1A-specific contigs were randomly selected and compared with the CS shotgun sequences. The sequence identity for this comparison was set at 70%, and all matching sequences larger than 50 bp were removed. The remainder CRNIL1A sequences with identify scores of less than 70% were used to calculate the total length and ratio of CRNIL1A sequences missing in CS.

### Validation of CRNIL1A-specific contigs

Primers designed for selected contigs were first assessed against the total DNA of CRNIL1A and CS (Supplementary Table S2). To minimize possible issues related to the ‘absence of a fragment’ in PCR reactions, DNA from CS was used as the negative control and that of CRNIL1A as the positive control in each of the PCR reaction. We also designed two sets of primers targeting four of the CS-missing fragments (Supplementary Table S2).

DNA was extracted from 20-day-old seedlings using the method of hexadecyl-trimethyl-ammonium bromide (CTAB)^39^. PCR amplification was performed in 10 μl reaction mixtures containing 50 ng of genomic DNA, 200 μM of each dNTP, 0.2 μM of each primer, and 0.5 units of Taq DNA polymerase. The cycling parameters were 94 °C for 5 min to pre-denature, which was followed by 35 cycles of 94 °C for 1 min, 30 s at the appropriate annealing temperature (ranging from 55 to 63 °C depending on the primers, see Supplementary Table S2), 72 °C for 1 min, and a final extension at 72 °C for 10 min. PCR products were separated on 2.0% agarose gels.

Primers that generated fragments differing in size between the two parents for either of the two segregating populations were used to genotype the population(s). Linkage analyses were conducted for all primer pairs that detected polymorphism in either of the populations. Linkage maps were generated using JoinMap 4^40^.

### Functional annotation of CRNIL1A-specific contigs

Functional annotation of the CRNIL1A-specific contigs was carried out using the Blast2GO^41^ as follows: the contig sequences were searched against GenBank NR protein database using the BLASTX algorithm with an E-value threshold of 10^−6^. The best 20 hits for each sequence were retained and used for GO-mapping. After GO terms had been mapped to each sequence, automated annotation was carried out using an annotation score cut-off of 55, an E-value threshold of 10^−6^, a GO weight of 5, and other parameters at default values. Gene ontology (GO) functional classification of all genes was further performed by choosing Ontology Level 2 for biological process, cellular component and molecular function in Blast2GO. It is well known that a single contig may contain several exons mapping to different proteins. To avoid biased results towards genes containing a larger number of exons, we counted the different proteins detected by a single contig once only in GO functional classification if they are similar in function.

### *De novo* assembly of hexaploid wheat transcripts

RNA sequencing data of 26 accessions of hexaploid wheat was downloaded from NCBI^42^. After trimming, high-quality reads were merged together and *de novo* assembled by Trinity^43^. The *de novo* assembled transcripts were then used as a reference to map back all the reads using RSEM^44^. We then filtered out any transcripts with less than 1% of the percomponent expression level (IsoPct) as these transcripts with low level of expression were likely to be artefacts of transcript assembly^45^. Those transcripts with FPKM value less than 0.1 were also removed from further assessment as they were defined as not expressed.

To detect novel transcripts not present in the CS genome, all the assembled transcripts were first checked by DeconSeq^38^. The filtered transcripts were aligned to CSS and gene models of CS on the IWGSC sequence assembly and TGACv1 using GMAP^46^. Those transcripts with alignments of >85% coverage and 85% identity were removed from further analysis. Considering the likely presence of alternative splicing, the retained transcripts were then clustered and duplicates removed using the software CDHIT-EST^47^ at 95% sequence similarity.

To examine the reliability of these novel transcripts, we compared them with wheat EST sequences (http://www.plantdb.org), CRNIL1A-specific contigs, and the genome assembly of the synthetic hexaploid wheat ‘W7984’^48^. The whole genome reads of the 16 Australian bread wheat varieties were downloaded from the Australian wheat varieties database^49^ and mapped to CS genome sequence by CLC Genomics Workbench. Those reads which could not be mapped to the CS genome were *de novo* assembled and treated as novel wheat genomic contigs. We then compared the novel transcripts with these wheat genomic contigs. Further, the novel transcripts were aligned to the whole genome shotgun (WGS) sequences of related species of wheat^3^,^50^,^51^. The software GMAP^46^ was used for comparing the transcripts with the genome sequences. Those transcripts with an alignment of >85% coverage and >85% identity were considered having counterparts in these genomic sequences.

## Additional Information

**How to cite this article**: Liu, M. *et al.* Chromosome-specific sequencing reveals an extensive dispensable genome component in wheat. *Sci. Rep.* **6**, 36398; doi: 10.1038/srep36398 (2016).

**Publisher’s note:** Springer Nature remains neutral with regard to jurisdictional claims in published maps and institutional affiliations.

## Supplementary Material

Supplementary Information

## Figures and Tables

**Table 1:** Summary of sequences obtained flow-sorted chromosome 3B of CRNIL1A.

Total	Raw sequence reads	~122.4 million
	High-quality reads	~74.0 million
High-quality reads mapped to CS 3B pseudomolecule	Number	~65.0 million
	Percentage	87.9%
	Mean read length	204 bp
	Coverage of 3B pseudomolecule length	692.7 Mb (89.5%)
	Average depth of coverage	17.1 times
High-quality reads not mapped to CS 3B pseudomolecule	Number	~9.0 million
	Percentage	12.1%

## References

[b1] Paux E. *et al.* Characterizing the composition and evolution of homoeologous genomes in hexaploid wheat through BAC-end sequencing on chromosome 3B. Plant J 48, 463–474 (2006). 17010109 10.1111/j.1365-313X.2006.02891.x

[b2] Choulet F. *et al.* Megabase level sequencing reveals contrasted organization and evolution patterns of the wheat gene and transposable element spaces. Plant Cell. 22, 1686–1701 (2010). 20581307 10.1105/tpc.110.074187 PMC2910976

[b3] International Wheat Genome Sequencing Consortium. A chromosome-based draft sequence of the hexaploid bread wheat (*Triticum aestivum*) genome. Science. 345, 1250092–1250092 (2014). 25035500 10.1126/science.1251788

[b4] Choulet F. *et al.* Structural and functional partitioning of bread wheat chromosome 3B. Science. 345, 1249721–1249721 (2014). 25035497 10.1126/science.1249721

[b5] Liu C. & Ogbonnaya F. C. Resistance to Fusarium crown rot in wheat and barley: a review. Plant Breeding. 134, 365–372 (2015).

[b6] Li H. B. *et al.* Genetic relationships between resistances to Fusarium head blight and crown rot in bread wheat (*Triticum aestivum* L.). Theor. Appl. Genet. 121, 941–950 (2010). 20535443 10.1007/s00122-010-1363-0

[b7] Ma J. *et al.* Identification and validation of a major QTL conferring crown rot resistance in hexaploid wheat. Theor. Appl. Genet. 120, 1119–1128 (2010). 20035314 10.1007/s00122-009-1239-3

[b8] Ma J., Yan G. J. & Liu C. J. Development of near-isogenic lines for a major QTL on 3BL conferring *Fusarium* crown rot resistance in hexaploid wheat. Euphytica. 183, 147–152 (2012).

[b9] Ma J. *et al.* Transcriptome and allele specificity associated with a 3BL locus for Fusarium crown rot resistance in bread wheat. PLoS ONE. 9, e113309 (2014). 25405461 10.1371/journal.pone.0113309 PMC4236173

[b10] Zheng Z. *et al.* Fine mapping of a large-effect QTL conferring *Fusarium* crown rot resistance on the long arm of chromosome 3B in hexaploid wheat. BMC Genomics. 16, 850 (2015). 26493707 10.1186/s12864-015-2105-0 PMC4618961

[b11] Cápal P., Blavet N., Vrána J., Kubaláková M. & Doležel J. Multiple displacement amplification of the DNA from single flow–sorted plant chromosome. Plant J 84, 838–844 (2015). 26400218 10.1111/tpj.13035

[b12] Brenchley R. *et al.* Analysis of the bread wheat genome using whole-genome shotgun sequencing. Nature. 491, 705–710 (2012). 23192148 10.1038/nature11650 PMC3510651

[b13] Ma J. *et al.* Extensive pericentric rearrangements in the bread wheat (*Triticum aestivum* L.) genotype ‘Chinese Spring’ revealed from chromosome shotgun sequence data. Genome Biol. Evol. 6, 3039–3048 (2014). 25349265 10.1093/gbe/evu237 PMC4255769

[b14] Ma J. *et al.* Sequence-based analysis of translocations and inversions in bread wheat (*Triticum aestivum* L.). PLoS ONE. 8, e79329 (2013). 24260197 10.1371/journal.pone.0079329 PMC3829836

[b15] Ma J. *et al.* Putative interchromosomal rearrangements in the hexaploid wheat (*Triticum aestivum* L.) genotype ‘Chinese Spring’ revealed by gene locations on homoeologous chromosomes. BMC Evol. Biol. 15, 37 (2015). 25880815 10.1186/s12862-015-0313-5 PMC4364500

[b16] Subbaiyan G. K. *et al.* Genome-wide DNA polymorphisms in elite *indica* rice inbreds discovered by whole-genome sequencing. Plant Biotechnol. J 10, 623–634 (2012). 22222031 10.1111/j.1467-7652.2011.00676.x

[b17] Jia G. *et al.* A haplotype map of genomic variations and genome-wide association studies of agronomic traits in foxtail millet (*Setaria italic*). Nat. Genet. 45, 957–961 (2014). 10.1038/ng.2673 23793027

[b18] Huang X. *et al.* Genomic analysis of hybrid rice varieties reveals numerous superior alleles that contribute to heterosis. Nat. Commun. 6, (2015). 10.1038/ncomms7258 PMC4327311 25651972

[b19] Lai K. *et al.* Identification and characterization of more than 4 million intervarietal SNPs across the group 7 chromosomes of bread wheat. Plant Biotechnol. J 13, 97–104 (2015). 25147022 10.1111/pbi.12240

[b20] Medini D., Donati C., Tettelin H., Masignani V. & Rappuoli R. The microbial pan-genome. Curr. Opin. Genet. Dev. 15, 589–594 (2005). 16185861 10.1016/j.gde.2005.09.006

[b21] Hogg J. S. *et al.* Characterization and modeling of the *Haemophilus influenzae* core and supragenomes based on the complete genomic sequences of Rd and 12 clinical nontypeable strains. Genome Biol. 8, 1 (2007). 10.1186/gb-2007-8-6-r103 PMC2394751 17550610

[b22] Lefébure T. & Stanhope M. J. Evolution of the core and pan-genome of *Streptococcus*: positive selection, recombination, and genome composition. Genome Biol. 8, 1 (2007). 10.1186/gb-2007-8-5-r71 PMC1929146 17475002

[b23] Tettelin H., Riley D., Cattuto C. & Medini D. Comparative genomics: the bacterial pan-genome. Curr. Opin. Microbiol. 11, 472–477 (2008). 19086349 10.1016/j.mib.2008.09.006

[b24] Kahlke T., Goesmann A., Hjerde E., Willassen N. P. & Haugen P. Unique core genomes of the bacterial family *vibrionaceae*: insights into niche adaptation and speciation. BMC genomics. 13, 179 (2012). 22574681 10.1186/1471-2164-13-179 PMC3464603

[b25] Gore M. A. *et al.* A first-generation haplotype map of maize. Science. 326, 1115–1117 (2009). 19965431 10.1126/science.1177837

[b26] Chia J. M. *et al.* Maize HapMap2 identifies extant variation from a genome in flux. Nat. Genet. 44, 803–807 (2012). 22660545 10.1038/ng.2313

[b27] Read B. A. *et al.* Pan genome of the phytoplankton *Emiliania* underpins its global distribution. Nature. 499, 209–213 (2013). 23760476 10.1038/nature12221

[b28] Hirsch C. N. *et al.* Insights into the maize pan-genome and pan-transcriptome. Plant Cell. 26, 121–135 (2014). 24488960 10.1105/tpc.113.119982 PMC3963563

[b29] Li Y. H. *et al.* *De novo* assembly of soybean wild relatives for pan-genome analysis of diversity and agronomic traits. Nat. Biotechnol. 32, 1045–1054 (2014). 25218520 10.1038/nbt.2979

[b30] Lu F. *et al.* High-resolution genetic mapping of maize pan-genome sequence anchors. Nat. Commun. 6, (2015). 10.1038/ncomms7914 PMC4411285 25881062

[b31] Yao W. *et al.* Exploring the rice dispensable genome using a metagenome-like assembly strategy. Genome Biol. 16, 1–20 (2015). 26403182 10.1186/s13059-015-0757-3 PMC4583175

[b32] Jin M. *et al.* Maize pan-transcriptome provides novel insights into genome complexity and quantitative. Sci. Rep. 6, 18936 (2016). 26729541 10.1038/srep18936 PMC4733048

[b33] Landry C. R., Oh J., Hartl D. L. & Cavalieri D. Genome-wide scan reveals that genetic variation for transcriptional plasticity in yeast is biased towards multi-copy and dispensable genes. Gene. 366, 343–351 (2006). 16427747 10.1016/j.gene.2005.10.042

[b34] Dunn B., Richter C., Kvitek D. J., Pugh T. & Sherlock G. Analysis of the *Saccharomyces cerevisiae* pan-genome reveals a pool of copy number variants distributed in diverse yeast strains from differing industrial environments. Genome Res. 22, 908–924 (2012). 22369888 10.1101/gr.130310.111 PMC3337436

[b35] Vrána J. *et al.* Flow sorting of mitotic chromosomes in common wheat (*Triticum aestivum* L.). Genetics. 156, 2033–2041 (2000). 11102393 10.1093/genetics/156.4.2033 PMC1461381

[b36] Šimková H. *et al.* Coupling amplified DNA from flow-sorted chromosomes to high-density SNP mapping in barley. BMC Genomics. 9, 294 (2008). 18565235 10.1186/1471-2164-9-294 PMC2453526

[b37] Cox M. P., Peterson D. A. & Biggs P. J. SolexaQA: At-a-glance quality assessment of Illumina second-generation sequencing data. BMC bioinformatics. 11, 1 (2010). 20875133 10.1186/1471-2105-11-485 PMC2956736

[b38] Schmieder R. & Edwards R. Fast identification and removal of sequence contamination from genomic and metagenomic datasets. PLoS ONE. 6, e17288 (2011). 21408061 10.1371/journal.pone.0017288 PMC3052304

[b39] Murray M. G. & Thompson W. F. Rapid isolation of high molecular weight plant DNA. Nucleic. Acids. Res. 8, 4321–4326 (1980). 7433111 10.1093/nar/8.19.4321 PMC324241

[b40] Van Ooijen J. JoinMap version 4.0: Software for the calculation of genetic linkage maps in experimental population. Kyazma B.V. Wageningen, Netherlands. (2006).

[b41] Conesa A. *et al.* Blast2GO: a universal tool for annotation, visualization and analysis in functional genomics research. Bioinformatics. 21, 3674–3676 (2005). 16081474 10.1093/bioinformatics/bti610

[b42] Cavanagh C. R. *et al.* Genome-wide comparative diversity uncovers multiple targets of selection for improvement in hexaploid wheat landraces and cultivars. Proc. Natl. Acad. Sci. USA 110, 8057–8062 (2013). 23630259 10.1073/pnas.1217133110 PMC3657823

[b43] Grabherr M. G. *et al.* Full-length transcriptome assembly from RNA-Seq data without a reference genome. Nat. Biotechnol. 29, 644–652 (2011). 21572440 10.1038/nbt.1883 PMC3571712

[b44] Li B. & Dewey C. N. RSEM: accurate transcript quantification from RNASeq data with or without a reference genome. BMC Bioinformatics. 12, 1–16 (2011). 21816040 10.1186/1471-2105-12-323 PMC3163565

[b45] Mortazavi A., Williams B. A., McCue K., Schaeffer L. & Wold B. Mapping and quantifying mammalian transcriptomes by RNA-Seq. Nat. methods. 5, 621–628 (2008). 18516045 10.1038/nmeth.1226 PMC13303166

[b46] Wu T. D. & Watanabe C. K. GMAP: a genomic mapping and alignment program for mRNA and EST sequences. Bioinformatics. 21, 1859–1875 (2005). 15728110 10.1093/bioinformatics/bti310

[b47] Li W. & Godzik A. Cd-hit: a fast program for clustering and comparing large sets of protein or nucleotide sequences. Bioinformatics. 22, 1658–1659 (2006). 16731699 10.1093/bioinformatics/btl158

[b48] Chapman J. A. *et al.* A whole-genome shotgun approach for assembling and anchoring the hexaploid bread wheat genome. Genome Biol. 16, 26 (2015). 25637298 10.1186/s13059-015-0582-8 PMC4373400

[b49] Edwards D. *et al.* Bread matters: a national initiative to profile the genetic diversity of Australian wheat. Plant Biotechnol. J 10, 703–708 (2012). 22681313 10.1111/j.1467-7652.2012.00717.x

[b50] Ling H. Q. *et al.* Draft genome of the wheat A-genome progenitor *Triticum urartu*. Nature. 496, 87–90 (2013). 23535596 10.1038/nature11997

[b51] Jia J. *et al.* *Aegilops tauschii* draft genome sequence reveals a gene repertoire for wheat adaptation. Nature. 496, 91–95 (2013). 23535592 10.1038/nature12028

