# Supplementary material for: Chromosome-specific sequencing reveals an extensive dispensable genome component in wheat
Source: Sci Rep. 2016 Nov 8;6:36398. doi: 10.1038/srep36398 (PMC5099574; doi:10.1038/srep36398)
Supplement: Supplementary Information [file srep36398-s1.pdf]

Supplementary Figures and Tables

Chromosome-specific sequencing reveals an extensive dispensable genome component in wheat

Miao Liu[1, 2], Jiri Stiller[1], Kateřina Holušová[3], Jan Vrána[3], Dengcai Liu[2], Jaroslav Doležel[3], Chunji Liu[1*, 4]

[1]CSIRO Agriculture, 306 Carmody Road, St Lucia, QLD 4067, Australia

[2]Triticeae Research Institute, Sichuan Agricultural University, Wenjiang, Chengdu 611130, China

[3]Institute of Experimental Botany, Centre of the Region Haná for Biotechnological and Agricultural Research, Šlechtitelů 31, CZ-78371 Olomouc, Czech Republic

[4]School of Plant Biology, The University of Western Australia, Perth, WA 6009, Australia

*Corresponding author

E-mail: chunji.liu@csiro.au

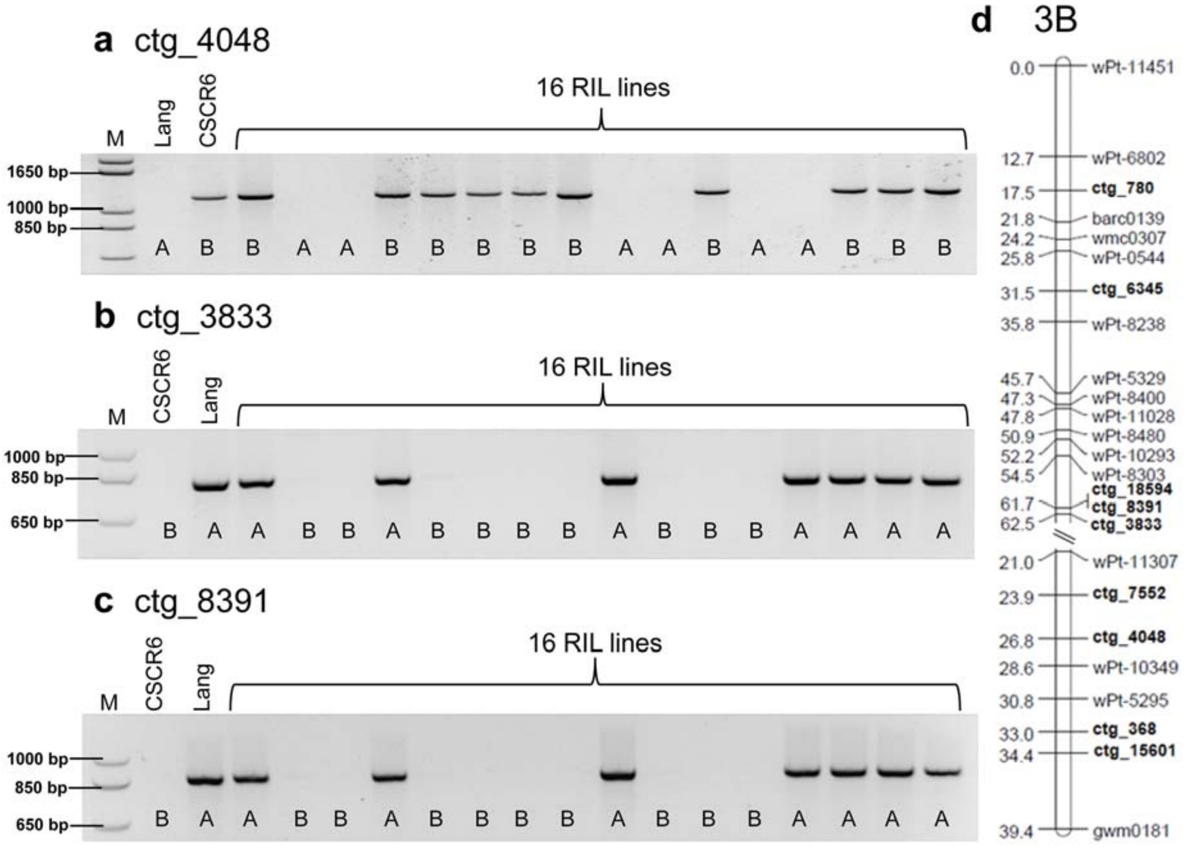

Supplementary Figure S1. Segregations of three (a, b & c) CRNIL1A-specific contigs (ctg) in a sample of the population of recombinant inbred lines Lang/CSCR6 (left) and the locations of nine contigs in the linkage map of chromosome 3B (right). M = marker, A = allele from the female parent Lang, and B = allele from the male parent CSCR6. Map positions were based on the linkage map published by Ma *et al.*[7].

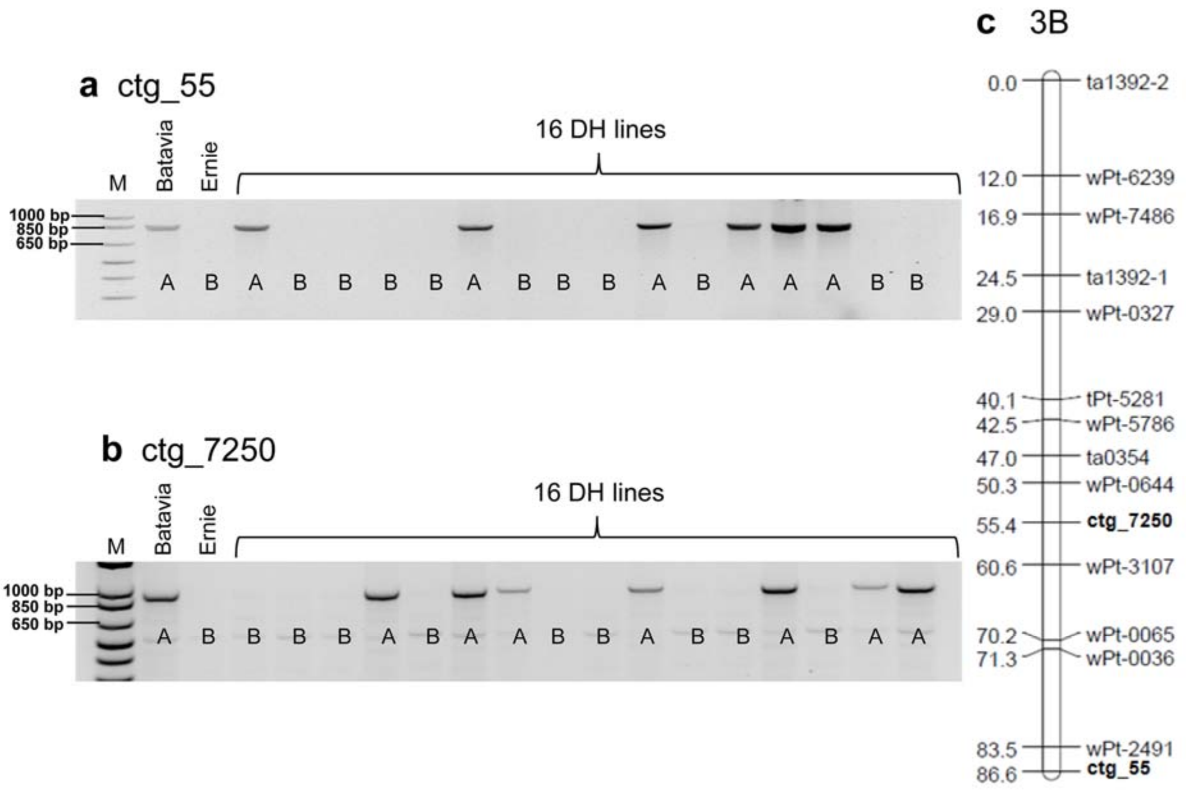

Supplementary Figure S2. Segregations of two (a & b) CRNIL1A-specific contigs (ctg) in a sample of the doubled haploid population Batavia/Ernie (left) and their locations in the linkage map of chromosome 3B (right). M = marker, A = allele from the female parent Batavia, and B = allele from the male parent Ernie. Map positions were based on the linkage map published by Li *et al.*[6].

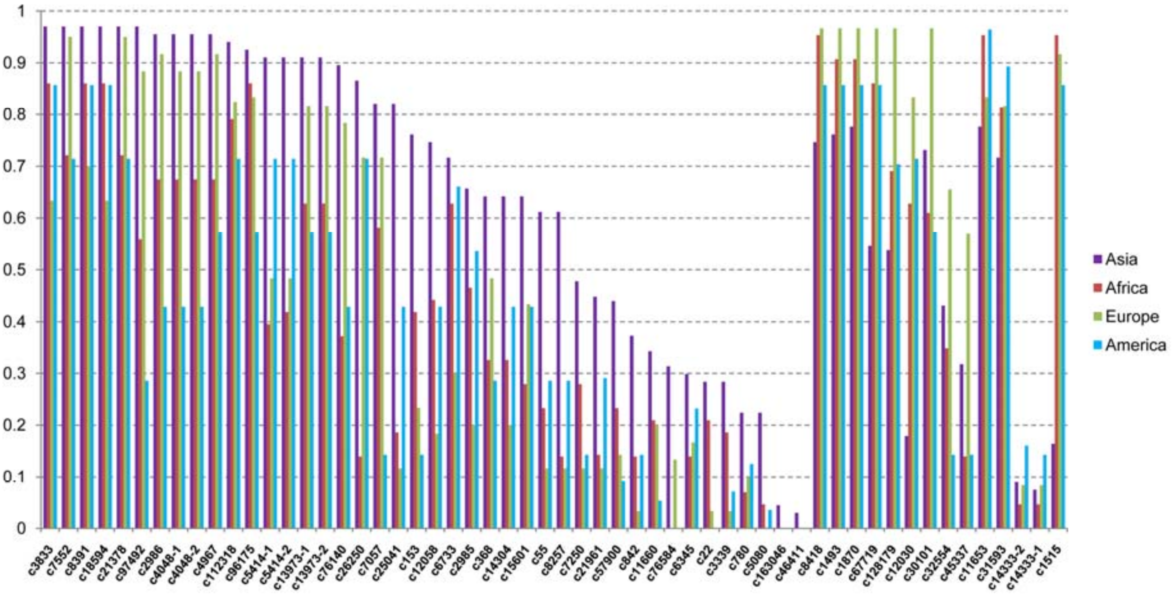

Supplementary Figure S3. Ratios (y-axis) of 'CS-type' alleles among the 226 hexaploid wheat genotypes assessed from the four different geographical regions assessed.

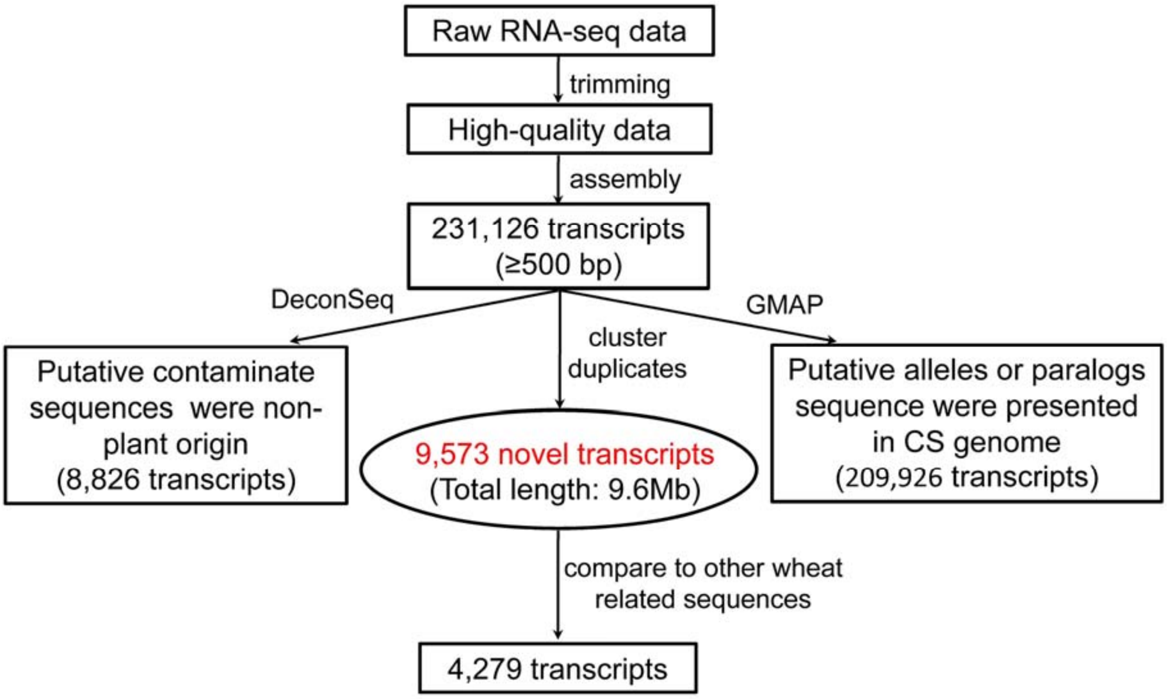

Supplementary Figure S4. Procedure of detecting and evaluating novel transcripts not present in CS.

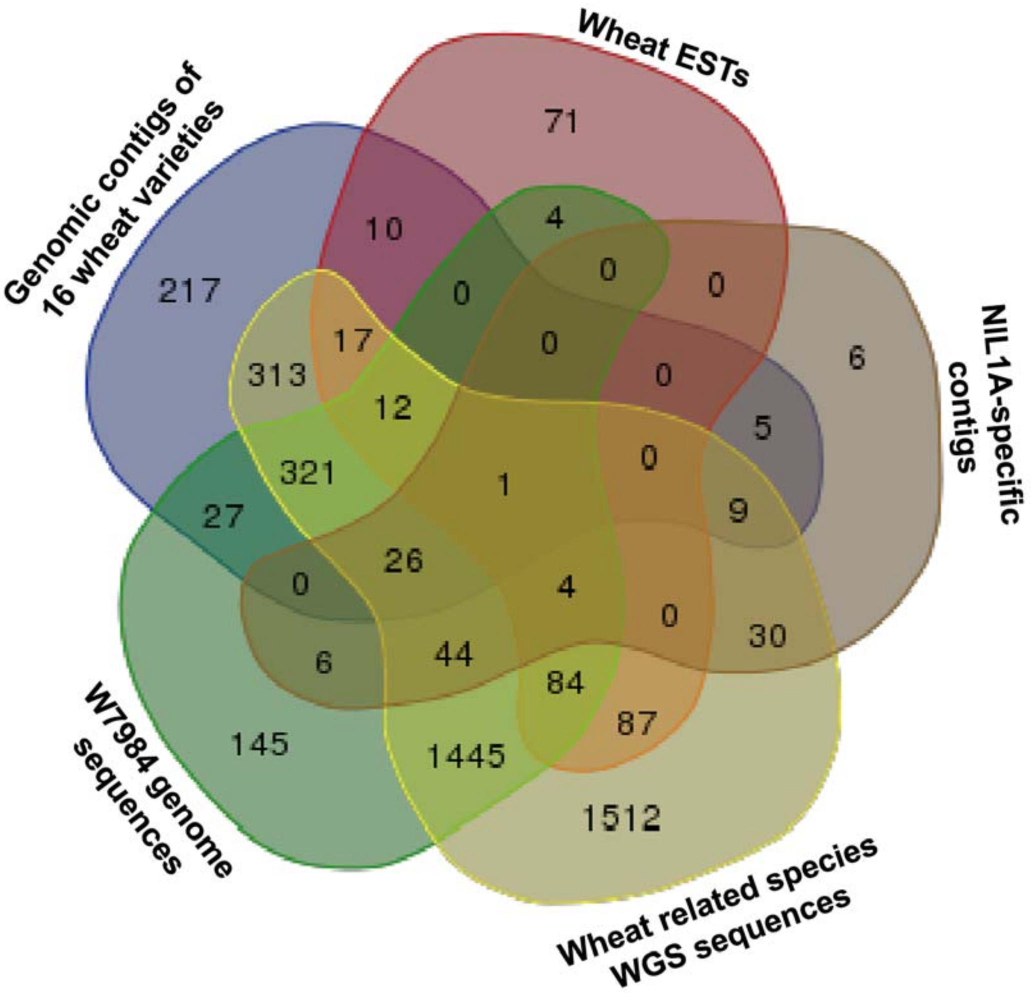

Supplementary Figure S5. Venn diagrams showing the number of novel transcripts matched with sequences of wheat and its close relatives.

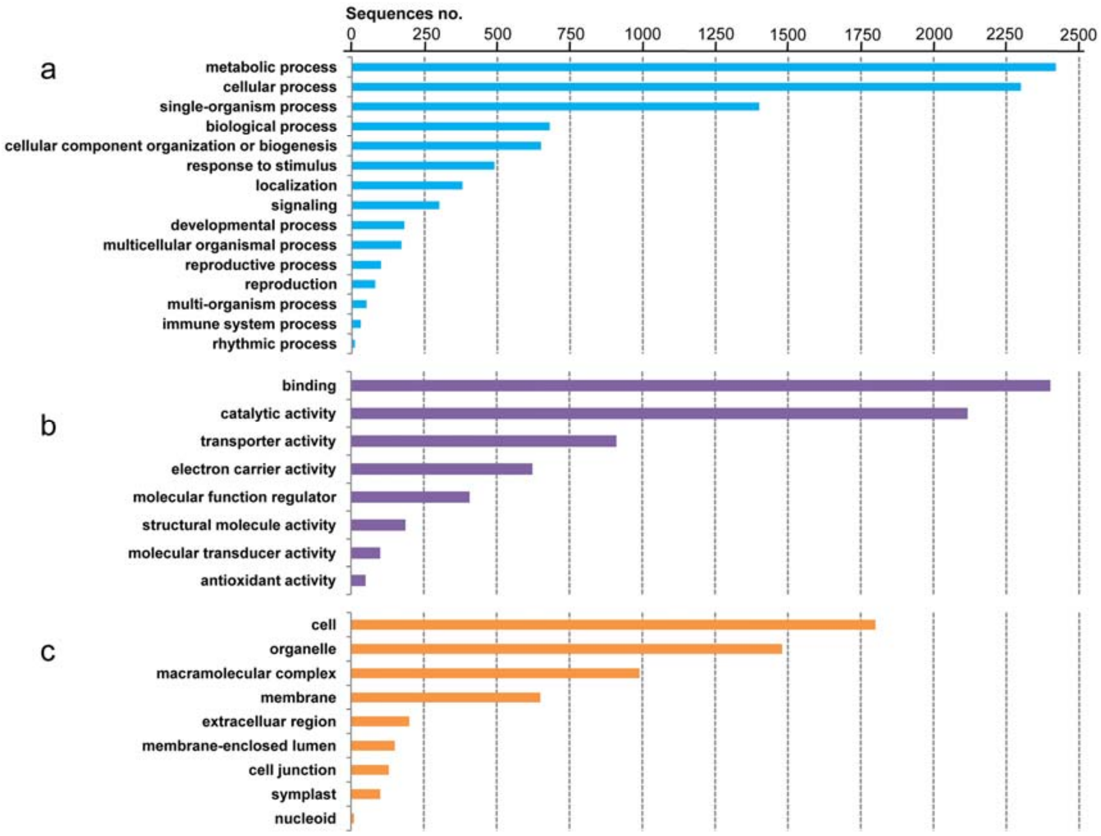

Supplementary Figure S6. Gene Ontology classification of genes represented by the 9,573 novel transcripts. Assignments of Gene Ontology terms for all sequences based on significant hits among various plant species are summarized into three main GO categories: (a) biological process, (b) molecular function, and (c) cellular component.

1

AUS.NO	Type	Origin
113	pedigree	CHINA
114	cultivar	CHINA
137	cultivar	MEXICO
239	cultivar	ITALY
240	cultivar	ITALY
243	cultivar	ITALY
244	cultivar	ITALY
347	cultivar	UNITED STATES
352	cultivar	UNITED STATES
387	cultivar	MEXICO
420	pedigree	ARGENTINA
423	cultivar	ARGENTINA
458	cultivar	MEXICO
643	cultivar	UNITED STATES
680	cultivar	MEXICO
688	cultivar	CHINA
699	collection	SPAIN
727	cultivar	MEXICO
761	cultivar	PAKISTAN
769	cultivar	MEXICO
829	Landrace	INDIA
831	Landrace	SOUTH AFRICA
859	cultivar	UNITED STATES
860	cultivar	UNITED STATES
1012	cultivar	CANADA
1180	cultivar	ITALY
1183	cultivar	JAPAN
1201	cultivar	INDIA
1305	cultivar	SOUTH AFRICA
1395	cultivar	MEXICO
1421	Landrace	PORTUGAL
1556	cultivar	UNITED STATES
1574	cultivar	FRANCE
1576	cultivar	FRANCE
1578	cultivar	ITALY
1579	cultivar	ITALY
1583	cultivar	ITALY
1695	cultivar	MEXICO
1725	cultivar	YUGOSLAVIA
1726	cultivar	YUGOSLAVIA
1739	cultivar	CHINA
1808	pedigree	MEXICO
1815	cultivar	ITALY
1818	cultivar	ITALY
1831	cultivar	ITALY

2004	cultivar	BRAZIL
2038	pedigree	MOROCCO
2048	cultivar	UNITED STATES
2126	pedigree	CHILE
2162	cultivar	BRAZIL
2164	cultivar	BRAZIL
2165	cultivar	BRAZIL
2191	cultivar	UNITED STATES
2564	cultivar	UNITED STATES
2731	cultivar	KENYA
2762	cultivar	ARGENTINA
2933	Landrace	EGYPT
2952	cultivar	MEXICO
2964	cultivar	CANADA
2995	cultivar	ITALY
3185	cultivar	CANADA
3273	cultivar	CANADA
4008	pedigree	CANADA
4101	collection	AFGHANISTAN
4102	collection	AFGHANISTAN
4103	collection	AFGHANISTAN
4104	collection	AFGHANISTAN
4201	cultivar	YUGOSLAVIA
4202	cultivar	YUGOSLAVIA
4570	pedigree	EGYPT
4639	collection	INDIA
4640	collection	INDIA
4643	collection	INDIA
4645	collection	INDIA
4655	pedigree	INDIA
4902	collection	IRAQ
4903	collection	IRAQ
4905	collection	IRAQ
4906	collection	IRAQ
4907	collection	IRAQ
4910	collection	IRAQ
4911	collection	IRAQ
4912	collection	IRAQ
4915	collection	IRAQ
4917	collection	IRAQ
4918	collection	IRAQ
4919	collection	IRAQ
5296	pedigree	PORTUGAL
5297	collection	PORTUGAL
5298	pedigree	PORTUGAL
5981	pedigree	KENYA
6008	pedigree	KENYA
6082	pedigree	MEXICO

6105	pedigree	MEXICO
6106	pedigree	MEXICO
6497	Landrace	INDIA
6621	pedigree	ARGENTINA
6622	pedigree	ARGENTINA
6938	Landrace	YUGOSLAVIA
6972	Landrace	TUNISIA
6980	Landrace	SPAIN
7054	Landrace	CHINA
7059	Landrace	CHINA
7061	pedigree	ARGENTINA
7062	pedigree	ARGENTINA
7279	cultivar	CHINA
7358	cultivar	CHINA
7439	cultivar	CHINA
7472	Landrace	JAPAN
7473	Landrace	JAPAN
7476	cultivar	MEXICO
7804	Landrace	AFGHANISTAN
7869	Landrace	AFGHANISTAN
7884	Landrace	SPAIN
7906	Landrace	TUNISIA
8556	Landrace	EGYPT
8626	Landrace	INDIA
8920	Landrace	YUGOSLAVIA
8921	Landrace	YUGOSLAVIA
9049	Landrace	SPAIN
10073	Landrace	TUNISIA
10462	cultivar	YUGOSLAVIA
10505	pedigree	CANADA
10636	cultivar	SOUTH AFRICA
10722	Landrace	SPAIN
10844	cultivar	HUNGARY
10895	cultivar	NETHERLANDS
10896	cultivar	SYRIA
11934	cultivar	CHILE
12007	pedigree	KENYA
12034	cultivar	MEXICO
12036	cultivar	MEXICO
12042	pedigree	ARGENTINA
12044	pedigree	ARGENTINA
12242	cultivar	IRAN
12298	cultivar	ARGENTINA
12347	cultivar	PORTUGAL
12548	cultivar	COLOMBIA
12583	pedigree	COLOMBIA
12584	pedigree	COLOMBIA
12631	pedigree	KENYA

12638	pedigree	KENYA
12726	pedigree	KENYA
13124	collection	MOROCCO
13129	Landrace	MOROCCO
13130	Landrace	MOROCCO
13131	Landrace	MOROCCO
13132	Landrace	MOROCCO
13133	Landrace	MOROCCO
13179	Landrace	TUNISIA
13182	Landrace	TUNISIA
13807	Landrace	AFGHANISTAN
13828	pedigree	SOUTH AFRICA
13832	pedigree	SOUTH AFRICA
13833	pedigree	SOUTH AFRICA
13835	pedigree	SOUTH AFRICA
13855	cultivar	IRAN
13906	cultivar	CHINA
13907	cultivar	CHINA
13908	cultivar	CHINA
13911	cultivar	CHINA
13917	cultivar	CHINA
13918	cultivar	CHINA
13921	cultivar	CHINA
14195	Landrace	ALGERIA
14345	pedigree	SWEDEN
14446	collection	AFGHANISTAN
14495	collection	AFGHANISTAN
14936	cultivar	ITALY
15137	Landrace	PORTUGAL
15164	Landrace	INDIA
15176	cultivar	BULGARIA
15659	cultivar	ITALY
15726	pedigree	ROMANIA
15727	pedigree	ROMANIA
15728	pedigree	ROMANIA
15729	pedigree	ROMANIA
15730	pedigree	ROMANIA
15731	pedigree	ROMANIA
16101	pedigree	BRAZIL
16356	pedigree	ROMANIA
16949	cultivar	ITALY
17210	cultivar	IRAN
17304	cultivar	TUNISIA
17484	cultivar	BULGARIA
17513	pedigree	MOROCCO
18368	pedigree	SOUTH AFRICA
18369	pedigree	SOUTH AFRICA
18433	cultivar	CANADA

18541	cultivar	BRAZIL
19068	Landrace	AFGHANISTAN
19459	Landrace	AFGHANISTAN
19468	Landrace	IRAN
19516	cultivar	ITALY
19524	cultivar	ITALY
19573	cultivar	SOUTH AFRICA
19598	cultivar	ZIMBABWE
19722	Landrace	EGYPT
20089	Landrace	JAPAN
20486	cultivar	ITALY
20537	Landrace	SAUDI ARABIA
20574	cultivar	EGYPT
20806	cultivar	ROMANIA
20839	pedigree	SOUTH AFRICA
20870	cultivar	KENYA
20870	cultivar	KENYA
21732	cultivar	SWITZERLAND
21738	cultivar	CHILE
22166	cultivar	FRANCE
23101	cultivar	KOREA, SOUTH
23102	cultivar	ROMANIA
23934	cultivar	ITALY
24467	cultivar	ROMANIA
24705	cultivar	HUNGARY
24927	Landrace	KYRGYZSTAN
25691	cultivar	CHILE
26904	Landrace	CHINA
26906	Landrace	CHINA
26907	Landrace	CHINA
27302	collection	COLOMBIA
27673	Landrace	ARMENIA
27674	cultivar	SWEDEN
30123	cultivar	ROMANIA
36464	Landrace	IRAQ
36467	Landrace	IRAQ
95153	pedigree	ROMANIA

Supplementary Table S1. Details of the 226 accessions of bread wheat genotypes selected to represent the four different geographical regions assessed.

Contig name	Forward primer (5'-3')	Revese primer (5'-3')	Expected/Product size (bp)	Annealing temperature (°C)
contig_22	CGGCGGTGGTGTGGTTAATTCC	AGAGGGAGGGAGGGAGGGTG	843	60
contig_55	TCCAGGGGAGAAATTGGGCT	TCTTGGGATGCATTGTGGGTG	861	60
contig_142	CGAGCGATGAAGGAGGAGGC	ACAGATTGTCGGTTGCAAGGC	962	FAILED
contig_153	CGCTCTCCGTCCAATTCCCTCA	ACCAATCCACACAACACGCTGGA	1236	60
contig_368	TGCATGGCTTCATAAACACCCT	ACCAACAACACCCTGTCCAACA	808	60
contig_780	GGCAAACTTCTCGGGCGGAA	GCCTCATTCGTGCCACCTCT	750	60
contig_842	TTCGCTGGTTGGGCTGTGCA	CCTTGCTGGGTTTCTGGGTGGT	1484	60
contig_1493	TGATGAGAATCAAATGGGCAAGGCA	TCTGACGGATGCTGAGTGAGGC	1376	60
contig_1515	AGCTCCCACAGTCCCATGCT	CTATGTGCCGCCCGTTGTGA	990	58
contig_1870	CCCCTTTAGACTGCGAGCCA	TCAGTCGGTCAGCTCTGGTG	910	63
contig_2985	GCAAGCGGGCAATCAAACCTCA	TCCGGTGCGATGTTGGACGT	1457	60
contig_2986	TGACAGACGTAGCCCCACATG	CAGGCGGTTCAACTGGGTCT	815	55
contig_3339	CCGCTTGATTTGTCAGATCCGATGA	TGCCCCGCGTAAAGTTTGGTG	1272	60
contig_3833	TCCATCTACAGGGAGCAGTCGT	GCCTTTGTGTTCCATGCGGTG	793	60
contig_4048-1	GGGGTCCTTGGAAGTGGTAAGTG	TCTCTGGCAAGCTATGGTAGGGA	1094	55
contig_4048-2	ACATGGGGATGGGCTTGACG	ATGTGTGCGTACTCTCCGGT	833	60
contig_4967	GCTTCATGTGACGTTCGTGT	CAAGCATCTGGACGATGGGA	946	58
contig_5080	AGTGCAACCCCTAGCTGTGT	TGAAGTGATGCGGACCTGGG	765	60
contig_5414-1	GTGATTAGACGCCAAGGGT	GGAAGTACAAAGCAACTCCATCA	610	60
contig_5414-2	GCAGCGCCTCGAACATTTTGC	CCGTCAGCAAACCCTTTCCTGA	1360	60
contig_5934	GGTTGGTGGGTGGGTTCTCG	GCTCAAATGTGCGGTGCGG	745	FAILED
contig_6345	TCATGAGTTTGGACCTTTGGA	TGGTTGTATGATGCTATGGTGTG	720	55
contig_7057	TGGACATCATGGCACTGTCCACAA	CCTCTAGTACCTACACTTGACCC	964	60
contig_7250	TGGCGCTTATACCCACAAG	CTGTAGCAGTTCCACCCTAC	885	55
contig_7552	GCAGCGAAAGGAGAAGAGGATAT	TCGTGCCATCCCATGAAGTACA	904	55
contig_8257	ACAGCAGGGGAGAGGCGAAA	ACGCAACAGGTCAAAGGAGTCAGT	1298	60
contig_8391	CAGTGTAATGCCCCTCCAGGT	AGCATCAATCCCAGCCAACAGA	854	60
contig_8418	TCCCCTTCAATGAGCCCCTCCA	ACCAGATGCTGCCACCGATGA	1405	60
contig_10390	CCGAAGCCCCTTTGCACTCA	TTTCATCCCGCCAACCACCC	964	FAILED
contig_10984	CTGCAAGCCGCATATTACCG	AATCCCCGAGCAGTTCATGG	700	55
contig_11660	CAGTTGTGCGGTGTGGAGCT	CAGCTCGGTGTGTTCGGGTT	1468	60
contig_11653	CCGTTCTTCATGTGGGCTTGGC	GCGTGTGTTGTGGTGTCAGTTGC	862	63

contig_13973-1	TGGCATTGGGAGGGTTGTGATTC	GGAAGAGAAGGCAAGGACGAGCA	1003	63
contig_13973-2	AGGGCTTCTAACATCCACCGCG	CACATCCAGCCTCAAACCAGCC	1030	63
contig_14304	GAGATCGTGGGGTCGCAGC	GTGTGGGGCCATTTCTCAAAGT	871	60
contig_14333-1	TGGGTTGTTTTGTGCAGCGC	ATGACCCTGGTAGCCGCAAC	1453	60
contig_14333-2	ACATGGGACTCGGAAAGA	GTAGAATGGGACGAAGGG	600	55
contig_15601	ATTCTACGTCTAGGAACG	CTTTAACGGATGTAGCAG	814	60
contig_18594	GCCTGAGACAAGGGGAAGGT	CGGTCAGGGAGGAGGAGATCA	976	60
contig_21378	GCTGCGATGGATTGTAAGGCC	GCTACGGGAGGAAAGGTCTAGC	1323	63
contig_26250	GTCCAAGAGAAGGCACCACTGG	ACCAACCCTTCCACCCACAGAC	831	63
contig_25459	TGAAGGTGGTGGAGGAGGAG	GCTGGGTCATATCTTGCGGTCTT	952	FAILED
contig_49487	CCATCCGAGTTGCTGCCCC	CAGGTTACGATACAAAAGGGCGA	657	FAILED

Supplementary Table S2. Details of primer pairs designed for CRNIL1A-specific contigs. Two pairs of primers (separated by a suffix '-1' or '-2') were used for four of these contigs while only a single one was designed for each of the other contigs.

Contig name	Forward primer (5'-3')	Revese primer (5'-3')	Expected/Product size (bp)		Annealing temperature (°C)
			NIL1A	CS	
contig_6733	TGATTGCAGCTCCCATGTGATTG	GGAGATGATGAGATGAGGCGAGG	468/468	269/269	55
contig_12030	AGGCTGAACTCAAGATGGGCGA	CGTGTCAAGTGCCGGAATGCC	1113/1113	806/806	64
contig_12058	ACTTGGCTCAACTGCTGACATTCA	AGCCACTTTTGTCCTTTTGCCTTCA	577/577	469/469	60
contig_12439	GCAGGGCTACATCTGGCGAA	GGCTTCGTGTTTCATCCGCC	585/585+463	463/463	58
contig_21961	GGTGTTGGCCCTGCAGTATCA	GACGCACGTTTCTCACAGTATACG	423/423	280/280	60
contig_25041	GTCCACAACTCCACCCCAGT	GGTCACCCTCTCCGCCATTG	699/699	608/608	60
contig_30101	CAATGGGGCTAGAAGGAGTAAAACA	GGTTGAGCGATATTAGGGATAGGGT	617/617	351/351	55
contig_31593	GGCTTCATTTCCGCTTGTAAAACA	TCATGTGTACTTGTCTACGGGCT	1049/1049	838/838	63
contig_32554	CGTGTGATGTTCGTATTCTAGGGGA	TCCAGCCCTCTTAATCCCACCA	300/300	230/230	60
contig_33506	GGACCGCTTGCATCATTGTCGT	TCTTGTCTTCTTCGCCACCGCT	1246/#	568/#	FAILED
contig_45337	TGTCAGAAACCAGAATCAGGAACAC	AGGACTGAAACGCTACCATCACA	1601/1601	235/235	60
contig_46411	GCGCCTGACTGATGCATTGC	ATTGTGCCACCCTTTTGCGC	1963/1963	673/673	60
contig_57900	GTGCCCGACAAGCCTACCAT	AGCCCCTTGATGCAGATCCTT	1036/1036	599/599	63
contig_67719	TGGCCCTTTAGTCAACACCCCT	CCCTACGGTTTCTAGCTAGCACACA	991/991	568/568	60
contig_76140	TCATGCCCTACCCTCCCTGT	TCCTCTCCACTAGCAGCACACA	574/574	461/461	60
contig_76584	GGCACCCGAACTATGGGCGA	GGAGGTCCCCATGTGAGGCC	1853/1853	836/836	64
contig_96175	GACGGAAAGCAGTAATCAATCGC	AAATGAGCCAAGGGAACAACATATG	452/452	343/343	55
contig_97492	CAGGGCTTTGGTTGGTGGGAA	ACAAGGGAAGTGGCGGAGATGT	967/967	724/724	63
contig_102975	CGCGCCATCTCAGCTTTCCT	TTCCCGACCGTCCGTTTCAG	627/627+425	425/425	60
contig_112318	CTGTGCTAGATGTCCACGAATCTGA	AGCTGTGAGGAAGATGGTTTGTCA	582/582	499/499	55
contig_117260	GACGTGGCTCTCTCTCGCGG	CTGCCTCCAACGCTGACCCA	2303/#	477/#	FAILED
contig_128179	TCAAATCACCCAGGAAAGCACACA	ACTCCATGCCCCTTCACGTTTTA	876/876	464/464	58
contig_143704	GCTCAGCAAAGAACCTCTGCTCG	AGTGGAAGATATACCTGGCCGTCT	2516/#	524/#	FAILED
contig_163046	GCACTCATAGGGGCAGCGTT	GCCACTAACTCCACGCACGT	882/882	652/652	63

Supplementary Table S3. Details of primer pairs targeting long insertions in CRNIL1A compared with CS. # indicates that no PCR product was detected.

Contig name	Allele type	Geographical origin			
		Asia	Africa	Europe	America
contig_22	CS	19	9	2	0
	CRNIL1A	48	34	58	56
contig_55	CS	41	10	7	16
	CRNIL1A	26	33	53	40
contig_153	CS	51	18	14	8
	CRNIL1A	16	25	46	48
contig_368	CS	43	14	29	16
	CRNIL1A	24	29	31	40
contig_780	CS	15	3	6	7
	CRNIL1A	52	40	54	49
contig_842	CS	25	6	2	8
	CRNIL1A	42	37	58	48
contig_1493	CS	51	39	58	48
	CRNIL1A	16	4	2	8
contig_1515	CS	11	41	55	48
	CRNIL1A	56	2	5	8
contig_1870	CS	52	39	58	48
	CRNIL1A	15	4	2	8
contig_2985	CS	44	20	12	30
	CRNIL1A	23	23	48	26
contig_2986	CS	64	29	55	24
	CRNIL1A	3	14	5	32
contig_3339	CS	19	8	2	4
	CRNIL1A	48	35	58	52
contig_3833	CS	65	37	38	48
	CRNIL1A	2	6	22	8
contig_4048-1	CS	64	29	53	24
	CRNIL1A	3	14	7	32
contig_4048-2	CS	64	29	53	24
	CRNIL1A	3	14	7	32
contig_4967	CS	64	29	55	32
	CRNIL1A	3	14	5	24
contig_5080	CS	15	2	0	2

	CRNIL1A	52	41	60	54
contig_5414-1	CS	61	17	29	40
	CRNIL1A	6	26	31	16
contig_5414-2	CS	61	18	29	40
	CRNIL1A	6	25	31	16
contig_6345	CS	20	6	10	13
	CRNIL1A	47	37	50	43
contig_7057	CS	55	25	43	8
	CRNIL1A	12	18	17	48
contig_7250	CS	32	12	7	8
	CRNIL1A	35	31	53	48
contig_7552	CS	65	31	57	40
	CRNIL1A	2	12	3	16
contig_8257	CS	41	6	7	16
	CRNIL1A	26	37	53	40
contig_8391	CS	65	37	42	48
	CRNIL1A	2	6	18	8
contig_8418	CS	50	41	58	48
	CRNIL1A	17	2	2	8
contig_11660	CS	23	9	12	3
	CRNIL1A	44	34	48	53
contig_11653	CS	52	41	50	54
	CRNIL1A	15	2	10	2
contig_13973-1	CS	61	27	49	32
	CRNIL1A	6	16	11	24
contig_13973-2	CS	61	27	49	32
	CRNIL1A	6	16	11	24
contig_14304	CS	43	14	12	24
	CRNIL1A	24	29	48	32
contig_14333-1	CS	5	2	5	8
	CRNIL1A	62	41	55	48
contig_14333-2	CS	6	2	5	9
	CRNIL1A	61	41	55	47
contig_15601	CS	43	12	26	24
	CRNIL1A	24	31	34	32
contig_18594	CS	65	37	38	48
	CRNIL1A	2	6	22	8

contig_21378	CS	65	31	57	40
	CRNIL1A	2	12	3	16
contig_26250	CS	58	6	43	40
	CRNIL1A	9	37	17	16
contig_6733	CS	48	27	18	37
	CRNIL1A	19	16	42	19
contig_12030	CS	12	27	50	40
	CRNIL1A	55	16	10	16
contig_12058	CS	50	19	11	24
	CRNIL1A	17	24	49	32
contig_21961	CS	30	6	7	16
	CRNIL1A	37	36	53	39
contig_25041	CS	55	8	7	24
	CRNIL1A	12	35	53	32
contig_30101	CS	49	25	58	32
	CRNIL1A	18	16	2	24
contig_31593	CS	48	35	49	50
	CRNIL1A	19	8	11	6
contig_32554	CS	28	15	38	8
	CRNIL1A	37	28	20	48
contig_45337	CS	20	6	33	8
	CRNIL1A	43	37	25	48
contig_46411	CS	2	0	0	0
	CRNIL1A	65	43	60	56
contig_57900	CS	29	10	10	5
	CRNIL1A	37	33	60	50
contig_67719	CS	36	37	57	48
	CRNIL1A	30	6	2	8
contig_76140	CS	60	16	47	24
	CRNIL1A	7	27	13	32
contig_76584	CS	21	0	8	0
	CRNIL1A	46	43	52	54
contig_96175	CS	62	37	50	32
	CRNIL1A	5	6	10	24
contig_97492	CS	65	24	53	16
	CRNIL1A	2	19	7	40
contig_112318	CS	63	34	47	40

	CRNIL1A	4	9	10	16
contig_128179	CS	36	29	58	38
	CRNIL1A	31	13	2	16
contig_163046	CS	3	0	0	0
	CRNIL1A	64	42	60	55

Supplementary Table S4. Numbers of the ‘CS-type’ and ‘CRNIL1A-type’ alleles detected by 56 pairs of primers among the 226 hexaploid wheat genotypes assessed.
